# Trends and determinants of diarrhea among under-five children in Ethiopia: cross-sectional study: multivariate decomposition and multilevel analysis based on Bayesian approach evidenced by EDHS 2000–2016 data

**DOI:** 10.1186/s12889-021-10191-3

**Published:** 2021-01-22

**Authors:** Yilkal Negesse, Asefa Adimasu Taddese, Ayenew Negesse, Tadesse Awoke Ayele

**Affiliations:** 1 Department of Epidemiology and Biostatistics, School of Public Health, College of Health Science, Mizan-Teferi, Ethiopia; 2 Department of Epidemiology and Biostatistic, Institute of public health, College of Medicine and Health Science, Gondar, Ethiopia; 3 Department of Human Nutrition and Food Science, College of Medicine and Health Science, Debre Markos, Ethiopia

**Keywords:** Ethiopia, Diarrhea, Demographic health surveillance, Under-fives, Children, Trends

## Abstract

**Background:**

Despite significant progress in the reduction of under-five child deaths over the last decades in Ethiopia, still diarrhea remains the second cause of morbidity and mortality among under five children next to pneumonia.

**Objective:**

To show trends and determinants of diarrhea among under five children in Ethiopia based on the four Ethiopian Demographic and health surveys data (2000–2016).

**Methods:**

A total of 10,753 in 2000, 10,039 in 2005, 10,946 in 2011 and 10,337 in 2016 under five age children were involved in this study. Multivariate decomposition and multilevel analysis based on Bayesian approach was performed.

**Results:**

Ninety seven percent of the change in diarrhea prevalence over time was attributable to difference in behavior. Being twin (AOR = 1.3; 95% CrI 1.1–1.5), big weight (AOR = 1.63; 95% CrI 1.62–2.02), not vaccinated for rotavirus (AOR = 1.44; 95% CrI 1.12–1.9) and for measles (AOR = 1.2; 95% CrI 1.1–1.33), poor wealth status (AOR 2.6; 95% CrI 1.7–4.06), having more than three under-five children (AOR 1.3; 95% CrI 1.1–1.61), member of health insurance (AOR 2.2; 95% CrI 1.3–3.8) and long distance from the health facility (AOR 2.7; 95% CrI 2.2–3.5) were more likely to experience diarrhea.

**Conclusion:**

The prevalence of diarrhea was significantly declined over the last sixteen years and the decline was due to difference in behavior between the surveys. Being twin, weight of child at birth, vaccinated for measles and rotavirus, number of under-five children, wealth status, distance to health facility, health insurance and child waste disposal method were significantly associated with diarrhea among under five children in Ethiopia. Therefore Ethiopian government should focus on the strengthening and scaling up of behavioral change packages of the community regarding to keeping hygiene and sanitation of the community and their environment, vaccinating their children, accessing health care services to prevent diarrheal disease.

## Background

According to World Health Organization (WHO) diarrhea is defined as passing loose or watery stool for three or more times during a 24-h’s period [1]. It is classified in to different categories based on different ways, but commonly classified as acute and persistent diarrhea [2–4]. Acute diarrhea is caused by an infection and usually begins within 12 h to 4 days after exposure and resolves within three to seven days; whereas Persistent diarrhea is a diarrhea with or without blood that begins acutely and lasts for ≥14 days [4, 5]. Despite significant worldwide progress in reduction of children death by diarrhea over time, still diarrhea remains the major cause of morbidity and mortality of children. It accounts for one-fourth of all childhood deaths annually across the Globe and in Africa, it is the third leading cause of mortality and were responsible for an estimated 333,000 children death [4, 6–9]. Different studies showed that, inadequate and unsafe water, lack of sanitation and poor hygiene practices are a complex issue for different pathogens and accountable for the occurrence of diarrheal diseases [10, 11]. Approximately 1.5 to 2.2 million people dies each year by diarrhea linked to poor sanitation, unsafe disposal of wastes, lack of awareness of good hygienic practices, not vaccinated for Rota virus and Measles and drinking contaminated water [12, 13]. It has also a detrimental impact on childhood growth and cognitive development beyond the cause of death [14]. Ethiopia introduced a new initiative Health Extension program (HEP) in 2002/03 as a means of providing a comprehensive, universal, equitable and affordable health service for the rural population on the base of promotive, preventive and basic curative services [15]. The program was provided as a 16 packages focusing on health promotion and education supported by demonstration targeting households, particularly mothers and women through house to house visits [15]. But the development of targeted approaches to address this burden has been hampered by a paucity of comprehensive, fine-scale estimates of factors related to diarrheal disease and death among and within countries [8].

Even though still morbidity and mortality of children due to diarrhea is high in Ethiopia; the prevalence of diarrhea among under five children decreased from 26% in 2000 to12% in 2016 [16]. The decrease in the prevalence of diarrhea could be explained by: (a) behavioral change (b) change of population proportion over time. The question of substantive interest in this context is: how much of the change is actually due to the improvement of behavior suggesting the actual decrease in diarrhea prevalence and how much is due to a compositional change in the population distribution. So to prevent and control diarrhea, it is necessary to know the trends of diarrhea over time, source of variation, the contributing factors for the change in prevalence of diarrhea precisely and determinants of diarrhea using appropriate statistical method of analysis. Because inappropriate result leads to misleading conclusion and intervention. But there is no evidence that shows studies conducted at national level to identify determinants of diarrhea by considering the clustering effect using Bayesian approach and that examine the contributing factors for the change in the prevalence of diarrhea among under-five children via decomposition analysis. Therefore, the aim of this study was to describe trends, identify the factors that contributed positively or negatively for the change in diarrhea prevalence among under five children for the last sixteen years and to identify determinants of diarrhea in Ethiopia based on data of 2016 Ethiopian Demographic and Health Survey.

## Methods

### Data source and population

We used 2000, 2005, 2011 and 2016 Ethiopian Demographic and Health surveys (EDHSs) data. These EDHSs are nationally representative cross-sectional surveys performed in 9 regions and 2 country city administrations every five years. In each of the surveys, stratified two-stage sampling of clusters was carried out. Stratification was achieved by separating each region into urban and rural areas. Accordingly, a total of 21 sampling strata have been created. In the first stage, a total of 539 Enumeration Areas (EAs) for EDHS 2000, 540 EAs for EDHS 2005, and 645 EAs for EDHS 2016 were randomly selected proportional to the EA size. At the second stage, on average 27 to 32 households per EA were selected [17–19]. The data was accessed from the Measure DHS website (http://www.dhsprogram.com) after permission was granted through an online request by explaining the objective of our study. The variables of the study were extracted from Kid Record (KR file) data set. Total weighted sample of 10,753, 10,039, 10,946 and 10,337 children used in EDHS 2000, 2005, 2011 and 2016 respectively for this study. The comprehensive procedure for sampling was described in the complete EDHS report [17–19].

### Variables

The outcome variable of this study was having diarrhea for the last two weeks prior to the data collection and coded as “Yes = 1” and “No = 0”. The EDHS asked respondents to answer the question “did your kid have diarrhea within those two weeks?” So, the response is a dichotomous with possible values *Y*_*i*_ = Yes if *i*^*th*^ child had diarrhea and *Y*_*i*_ = No if the child had no diarrhea.

The independent variables were classified as community and individual level variables. Place of residence and region of the study participants were considered as community level variables. Whereas family size, number of under-five children, educational status of both husband and mother, working status of mother, wealth index of parents, media exposure, distance to health facility, health insurance, age of the child, sex of the child, being twin, weight of the child at birth, breast feeding, vaccinated for rotavirus and measles, vitamin A supplementation, type of drinking water source, type of latrine and way of child waste disposal were considered as individual level factors.

### Statistical analysis

The data were extracted from the KID Record (KR file) data sets. Before any statistical analysis, the data were weighted using sampling weight for probability sampling and non-response to restore the representativeness of the survey and get reliable statistical estimates.

### Trend and decomposition analysis

The trend period was divided into four phases; first phase (2000–2005), second phase (2005–2011), third phase (2011–2016) and the overall or fourth phase (2000–2016) to see the differences in diarrhea prevalence over time based on different characteristics. The trend was assessed using descriptive analyses stratified by different characteristics and was assessed separately for the periods 2000–2005, 2005–2011, 2011–2016, and 2000–2016.The multivariate decomposition analysis is a statistical analysis for examining the change in event that results in differences in outcome between any two surveys [20]. The aim of using decomposition analysis was to compare the difference in two time periods and identify the sources of variations of diarrhea prevalence among under five children. The difference between any two surveys was explained by the compositional changes or characteristics of surveys (endowments), which is explained and by the effects of those characteristics (coefficients) that is not explained [20]. Therefore, the observed change in burden of diarrhea between two surveys was additively decomposed in the endowment (characteristics) component and coefficient (effect of characteristics) component using recently developed **mvdcmp** Stata package. In the nonlinear model, the response variable is a function of a linear combination of predictors and regression coefficients [20].

$$ \mathrm{Y}=\mathrm{F}\left(\mathrm{X}\upbeta \right)=\mathrm{logit}\left(\mathrm{Y}\right)=\mathrm{X}\upbeta $$

Where *Y* represent the dependent variable.

*X* represents a set of predictor variables.

*β* denote set of regression coefficients.

The proportion difference in Y between the two surveys of A and B can be decomposed as

Y_A_ − Y_B_ = F(X_A_β_A_) − (X_B_β_B_) [20].

Let the recent 2016 EDHS and reference 2000 EDHS datasets can be denoted by A and B respectively.

For logistic regression, the log-odds or logit of the burden of diarrhea is given by [20].

$$ \mathrm{logit}\left(\mathrm{A}\right)-\mathrm{logit}\left(\mathrm{B}\right)=\mathrm{F}\left({\mathrm{X}}_{\mathrm{A}}{\upbeta}_{\mathrm{A}}\right)-\mathrm{F}\left({\mathrm{X}}_{\mathrm{B}}{\upbeta}_{\mathrm{B}}\right) $$

= [*F*(*X*_*A*_*β*_*A*_) − *F*(*X*_*B*_*β*_*A*_)] + [*F*(*X*_*B*_*β*_*A*_) − *F*(*X*_*B*_*β*_*B*_]

E $$ C $$

Where; *E* represents endowments, which is explained by characteristics. An endowment is a change in diarrhea due to differences in characteristics. *C* denotes coefficients or effect of characteristics which is unexplained (20). The coefficient is the change in diarrhea due to the effect of predictor variables.

The equation can be presented as:

$$ \mathrm{logit}(A)-\mathrm{logit}(B)=\left[{\beta}_{0A}-{\beta}_{0B}\right]+\sum {X}_{ijB}\ast \left[{\beta}_{ijA}-{\beta}_{ijB}\right]+\sum {\upbeta}_{ijB}\ast \left[{X}_{ijA}-{X}_{ijB}\right] $$

Where; $$ {\beta}_{0B}\ \mathrm{is}\ \mathrm{the}\ \mathrm{in}\mathrm{tercept}\ \mathrm{in}\ \mathrm{the}\ \mathrm{regression}\ \mathrm{equation}\ \mathrm{for}\ \mathrm{EDHS}\ 2000 $$

*β*_0*A*_ is the intercept in the regression equation for EDHS 2016.

$$ {\beta}_{ijB}\ \mathrm{is}\ \mathrm{the}\ \mathrm{coefficient}\ \mathrm{of}\ \mathrm{the}\ {j}^{th}\ \mathrm{category}\ \mathrm{of}\ \mathrm{the}\ {i}^{th}\ \mathrm{determinant}\ \mathrm{in}\ \mathrm{EDHS}\ 2000 $$

$$ {\beta}_{ijA}\mathrm{is}\ \mathrm{the}\ \mathrm{coefficient}\ \mathrm{of}\ \mathrm{the}{j}^{th}\ \mathrm{category}\ \mathrm{of}\ \mathrm{the}\kern0.50em {i}^{th}\mathrm{determinant}\ \mathrm{in}\ \mathrm{EDHS}\ 2016 $$

$$ {X}_{ijB}\ \mathrm{is}\ \mathrm{the}\ \mathrm{proportion}\ \mathrm{of}\ \mathrm{the}\ {j}^{th}\ \mathrm{category}\ \mathrm{of}\ \mathrm{the}\kern0.50em {i}^{th}\ \mathrm{determinant}\ \mathrm{in}\ \mathrm{the}\ \mathrm{EDHS}\ 2000 $$

$$ {X}_{ijA}\ \mathrm{is}\ \mathrm{the}\ \mathrm{proportion}\ \mathrm{of}\ \mathrm{the}\ {j}^{th}\ \mathrm{category}\ \mathrm{of}\ \mathrm{the}\kern0.50em {i}^{th}\ \mathrm{determinant}\ \mathrm{in}\ \mathrm{EDHS}\ 2016 $$

To determine the specific contribution of each independent variable to each component of differences in the burden of diarrhea we partitioned the endowment and coefficients denoted by *C* and *E* into a portion of *C*_*k*_ and *E*_*k*_, which represent the specific contribution of *K*^*th*^ independent variables for each component of *C* and *E* respectively.

### Multilevel analysis based on Bayesian approach

To see the relationship between diarrhea and explanatory variables we applied multilevel binary logistic regression based on Bayesian approach evidenced by EDHS 2016 (the most recent) data. In this study, two levels of data hierarchy was stated. Level one unit were individual children of households and level two units were enumeration areas. Level one (children in the household) are nested within units at the next higher level (enumeration areas). The outcome variable was represented by *Y*_*ij*_ = $$ \left\{\begin{array}{c} having\ diarrhea\\ {} no\  diarrhea\ \end{array}\right. $$, the category is binary type of data. Therefore multilevel binary logistic regression analysis based on Bayesian approach was performed using **Brms** R-package to estimate the parameters of the variable and the extent of random variations between clusters.

Bayesian analysis approach is one of the data analysis approach independent to the classical analysis approach and the parameters are estimated from the posterior distribution which is the combination of the prior information and the likelihood of the data [21]. For this study we used vague prior with beta distribution [1] to estimate regression coefficients and gamma distribution (0.001, 0.001) to estimate the variance, iteration = 10,000 warmup =1000 (number of iterations that was discarded), chains =2, initials (the starting values of the iterations) =0, cores (specifies the number of cores used for the algorithm) =2 and adapt delta (controls divergent transition) = 0.95.

After posterior distribution was determined, we used No-U-Turn Sampler (NUTS) methods to simulate direct draws from the complex posterior distribution. No-U-Turn Sampler (NUTS) avoids the random walk behavior and sensitivity to correlated parameters that plague many MCMC methods by taking a series of steps informed by first-order gradient information [22]. .

Lastly four models were fitted and compared based on their Widely Applicable Information Criteria (WAIC) and Leave-One-Out Cross-Validation (LOO) value. A model with small WAIC and LOO is best model [23]. So, a model with small WAIC and LOO value was selected and all interpretations and inferences were made based on this model. We used the ICC value greater than 10% to consider variation of diarrhea prevalence across the cluster. For test of significance we used the 95% posterior credible interval in which the interval containing 1 is considered as non-significant.

The results obtained from a given HMC analysis are not deemed reliable until the chain has reached its stationary distribution [24]. Therefore, to monitor the convergence of the algorithm we used the most popular and straight forward convergence assessment methods in which Rhat =1, Bulk_ESS and Tail_ESS were greater than 1000, chains of the time serious plots were mixed well and density plot were smooth.

## Results

### Characteristics of the study population

Based on socio-demographic reports of EDHS data, more than 85% of the households were rural settlers. More than four fifth; 86.7% in 2000, 89.2% in 2005, 85.2% in 2011 and 86.2% of the total households in 2016 were leaded by males. With regard to education, the proportion of maternal higher educational status was 0.2% in 2000, 0.4% in 2005, 1.5% in 2011 and 2.5% in 2016 (Table 1).

**Table 1:** Frequency and Percentage distribution of characteristics of respondents and their children in Ethiopia

**Variables**	**Characteristics**	**Frequency and percentage distribution of characteristics**			
		EDHS 2000 No (%)	EDHS 2005 No (%)	EDHS 2011 No (%)	EDHS 2016 No (%)
**Sex of child**	**Male**	5460(51)	5089 (51)	5636(51.5)	5307(51)
	**Female**	5293(49)	4950 (49)	5310 (48.5)	5030(49)
**Twin**	**Yes**	136 (1)	143 (1.4)	203(1.9)	292 (2.2)
	**No**	10,617(99)	19,896(98.6)	10,743(98.1)	10,105 (97.8)
**Weight of child at birth**	**Small**	3651 (34)	2805 (28)	3251(29.7)	2676 (26.1)
	**Average**	388 (36)	4069 (40.1)	4275 (39.1)	4328 (42.2)
	**Big**	3200(30)	3149 (31.4)	3406 (31.2	3255 (31.7)
**Birth order**	**First**	1982 (19)	1666 (16.6)	2068 (18.9)	1923 (18.6)
	**2–3**	3264(30)	3069 (30.6)	3422 (31.3)	3155 (30.5)
	**4–5**	2362 (22)	2382 (23.7)	2522 [23]	2473 (23.9)
	**= > 6**	3144(29)	2920 (29.2)	2935 (26.8)	2786 (27)
**Birth interval**	**= < 23 month**	2215 (23)	3420 (34)	2407 (25)	2446 (27.3)
	**= > 24 month**	7951(77)	6618 (66)	7221 (75)	6499 (72.7)
**Age of child**	**< 1 year**	2186(20.3)	222 (22)	2383 (21.8)	2264 (21.9)
	**= > 1 < 2 year**	2145 (20)	1872 (18.7)	1915 (17.5)	2001 (19.4)
	**= > 2 < 3 year**	2084(19.4)	1883 (18.8)	2045 (18.7)	1926(18.6)
	**= > 3 < 4 year**	2260 (21)	2078(20.7)	2351 (21.5)	1980 (19.1)
	**= > 4 < 5 year**	2080(19.3)	1984 (19.8)	2251 (20.6)	2165 (21)
**Breast feeding**	**Still feeding**	5318 (49.5)	5111(51.2)	5253 (45)	4325 (42)
	**Ever feed but not now**	5366 (50)	4699 (47)	5532 (50.5)	5624 (54.4)
	**Never feed**	69 (0.64)	178 (4.1)	161 (1.5)	388 (3.8)
**Rotavirus vaccine**	**vaccinated**	–	–	–	2858 (27.7)
	**Not vaccinated**	–	–	–	7479 (72.4)
**Measles’ vaccine**	**Vaccinated**	2894 (26.9)	2535 (26.9)	5053 (47.1)	3692 (60.2)
	**Not vaccinated**	7859 (73.1)	6877(73.1)	5682(52.9)	2446(39.8)
**Vitamin A supplemented**	**Yes**	6000(56.5)	4399(44.5)	5274 (48.8)	4574 (48.7)
	**No**	4624(43.5)	5492 (55.5)	5540 (51.2)	4825 (51.3)
**Wealth index**	**Poor**	–	4312(35)	4889 (44.7)	4848(46.9)
	**Medium**	–	2197 (22)	2259 (20.6)	2139 (20.7)
	**Rich**	–	3529 (35)	3798 (34.7)	3350 (32.4)
**Mothers work status**	**Had work**	6003 (55.9)	8572 (76.8)	3733 (34.1)	2803 (27.1)
	**had no working**	4745(44.1)	2590 (23.2)	4061 (68.9)	7534 (72.9)
**Health insurance**	**Yes**	–	–	59 (0.5)	378 (3.7)
	**No**	–	–	10,882(99.4)	9959 (96.3)
**Distance to health facility**	**Not long**	–	2553 (25.4)	2710(24.8)	4056 (39.2)
	**Long**	–	7485 (74.6)	8227 (75.2)	6281 (60.8)
**Continuation of above table**					
**Variables**	**Characteristics**	**Frequency and percentage distribution of characteristics**			
		EDHS 2000 No (%)	EDHS 2005 No (%)	EDHS 2011 No (%)	EDHS 2016 No (%)
**Residence**	**Urban**	1141(10.6)	741(7.4)	1413(12.9)	1151 (11.1)
	**Rural**	9612(89.4)	2298(92.6)	9534(87.1)	9186 (88.9)
**Region**	**Tigray**	709 (6.6)	652 (6.5)	701 (6.3)	682 (6.6)
	**Afar**	107 (1)	96 (1)	110 (1)	104 (1)
	**Amhara**	2797 (26)	2299 (22.9)	2472 (22.6)	1954 (18.9)
	**Oromia**	4356 (40.5)	3982 (39.7)	4615(42.2)	4537 (43.9)
	**Somali**	127(1.2)	428 (4.3)	330 (3)	472 (4.6)
	**B/Gumuz**	108 (1)	95 (0.9)	125(1)	113 (1.1)
	**SNNP**	2297 (21.4)	2265 (22.6)	2287 (20.9)	2149 (20.8)
	**Gambella**	25(0.23)	28 (0.3)	35 (0.3)	24 (0.2)
	**Harari**	23 (0.2)	21 (0.2)	26 (0.2)	24 (0.2)
	**Addis Ababa**	167 (1.6)	140 (1.4)	208 (1.9)	233 (2.3)
	**Dire-Dawa**	35 (0.3)	34 (0.3)	36 (0.3)	43(0.4)
**Mother’s educational level**	**No formal education**	8771 (82)	7896(78.7)	7562 (69.1)	6809 (65.9)
	**Primary**	1426 (13)	1699 (16.9)	2980 (27.2)	2777(26.9)
	**Secondary**	532 (4.9)	401 (4)	245(2.2)	491 (4.8)
	**Higher**	24 (0.2)	42 (0.4)	159(1.5)	260 (2.5)
**Father’s educational level**	**No formal education**	6732 (63.5)	5771(58.1)	5360 (49.7)	4700 (48.2)
	**Primary**	2682 (25.3)	3054 (30.8)	4527(42)	3849 (39.5)
	**Secondary**	1054 (10)	1000 (10.1)	550(5.1)	766 (7.9)
	**Higher**	140 (1.3)	111 (1.1)	348 (3.2)	442 (4.5)
**Sex of household head**	**Male**	9327(86.7)	8957 (89.2)	9323 (85.2)	8908 (86.2)
	**Female**	1426(13.3)	1082(10.8)	1623 (14.8)	1429 (13.8)
**Media exposure**	**Yes**	893(8.3)	1222 (12.2)	2007(18.4)	1846 (17.9)
	**No**	9849(91.7)	8799 (87.8)	8927(81.6)	8491 (82.1)
**Family members**	**= < 5**	4655 (43.3)	4116 (41)	4812(44)	5865 (56.7)
	**= > 6**	6098 (56.7)	5922 (59)	6134 (56)	4472 (43.3)
**Number of U-5 children**	**= < 2**	9169 (85.3)	5922 (83.2)	9041 (82.6)	8541 (82.6)
	**= > 3**	1584 (14.7)	1685 (16.8)	1906 (17.4)	1796 (17.4)
**drinking water**	**Improved**	2224(21.4)	5289(55.4)	4951 (46.5)	5719 (56.2)
	**Not improved**	8169(78.6)	4254(44.6)	5686 (53.5)	4464 (43.8)
**Child waste disposal**	**Safe**	2457 (23)	1987 (20)	3647(36.2)	2553 (38.8)
	**Not safe**	8256 (77)	7945 (80)	6419 (63.8)	4035 (61.2)
**Type of toilet**	**Improved**	1494(14.4)	891(9)	1342(12.6)	1036 (21.4)
	**Not improved**	8903(85.6)	9073(91)	9287 (87.4)	3804 (78.6)

According to birth related reports of EDHS data, the highest percentage (2.2%) of twin birth was reported in 2016. Both the highest and lowest proportion of small birth weight (34%) and (26.1%) was reported in 2000 and 2016 respectively. The highest proportion of narrow birth interval (<= 23 months) was reported in 2005 (34%) whereas the smallest proportion (23%) was also reported in 2000 (Table 1).

The proportion of children vaccinated for measles was 26.9% both in 2000 and 2005, 47% in 2011 and 60.7% in 2016. Though there was no reports about rotavirus vaccine in the first three EDHS’s, the last EDHS report showed that only 27.7% of children were vaccinated. Amongst the surveyed households, 77% in 2000, 80% in 2005, 63.8% in 2011 and 61.2% of households practiced unsafe way of infant waste disposal (Table 2).

**Table 2:** Trends of diarrhea prevalence among under-five children by selected characteristics in Ethiopia

						Point difference in diarrhea prevalence			
**Variables**	**Characteristics**	2000 N = 10,753	2005 N = 10,039	2011 N = 10,946	2016 N = 10,337	Phase1 (2005–2000)	Phase2 (2011–2005)	Phase3 2016–2011	Over all (2016–2000)
**Sex of child**	**Male**	26.3	18.1	14.4	12.2	−8.2	−4.1	−2.2	−14.1
	**Female**	24.9	18.1	12.6	11.5	−6.8	−5.5	−1.1	−13.4
**Twin**	**Yes**	19.3	14.8	23.6	13.8	−4.5	4.3	−5.5	−7.5
	**No**	25.7	18.2	13.4	11.8	−7.5	−4.8	−1.6	−13.9
**Weight of child at birth**	**Small**	26.3	20	14.9	15.7	−6.3	−5.9	0.8	−10.6
	**Average**	23.3	15.5	12.6	9.8	−7.8	−2.9	−2.8	−13.5
	**Big**	27.5	19.8	13.5	11.6	−7.7	−6.3	−1.9	− 15.9
**Birth order**	**First**	27.2	17.5	11.2	13.1	−9.7	−6.3	1.9	−14.1
	**2–3**	26.6	16.7	13.4	12.4	−9.9	−3.3	−1	14.2
	**4–5**	25.4	20.9	14.7	12	−4.5	−6.2	−2.7	−13.4
	**= > 6**	23.8	17.8	14.4	10.4	−6	−3.4	−4	−13.4
**Birth interval**	**= < 23 month**	25.2	17.6	10.4	9.9	−7.6	−7.2	−0.5	−15.3
	**= > 24 month**	24.9	18.4	14.3	11.6	−6.5	−4.1	−2.7	−13.3
**Age of child**	**< 1 year**	27.3	21.3	17.2	14.7	−6	−4.1	−2.5	−12.6
	**= > 1 < 2 year**	37.2	28.4	22.8	17.8	−8.8	−5.6	−5	−19.4
	**= > 2 < 3 year**	28.7	18.6	14.1	13	−10.1	− 4.5	− 1.1	− 15.7
	**= > 3 < 4 year**	19.2	12.6	9	9.2	−6.6	−3.6	0.2	−10
	**= > 4 < 5 year**	15.7	10.2	6	4.8	−5.5	−4.2	−1.2	− 10.9
**Breast feeding**	**Still feeding**	31.2	23.2	18.4	12	−8	−4.8	−6.4	−19.2
	**Ever feed but not now**	20	12.7	9.1	11.9	−7.3	−3.6	2.8	−8.1
	**Never feed**	36.2	19.1	13.5	10.3	−17.1	−5.6	−3.2	− 25.9
**Rotavirus vaccine**	**Vaccinated**	–	–	–	12.5	–	–	–	–
	**Not vaccinated**	–	–	–	11.6	–	–	–	–
**Measles’ vaccine**	**Vaccinated**	22.6	20	14	13.2	−2.6	−6	−0.8	−9.4
	**Not vaccinated**	26.7	17.7	13.3	18.2	−9	−4.4	4.9	−8.5
**Vitamin A supplemented**	**Yes**	24.9	19.4	13.6	12.8	−5.5	− 5.8	−0.8	− 12.1
	**No**	25.5	17.3	13.3	11.4	−8.2	−4	−1.9	− 14.1
**Mothers work status**	**had work**	26.7	18.5	13.2	11.3	−8.2	−5.3	−1.9	− 15.4
	**had no work**	24.8	18	14.1	13.3	−6.8	−3.9	−0.8	−11.5
**Wealth index**	**Poor**	–	19.1	13.8	12.6	–	−5.3	−1.2	–
	**Medium**	–	19.8	13.2	12.5	–	−6.6	−0.7	–
	**Rich**	–	15.9	13.5	12.6	–	−2.4	−0.9	–
**Health insurance**	**Yes**	–	–	10.3	10.8	–	–	0.5	–
	**No**	–	–	13.6	11.9	–	–	−1.7	–
**Distance to health facility**	**Not long**	–	16.3	12.1	12.6	–	−4.2	0.5	–
	**Long**	–	18.7	14	11.4	–	−4.7	−2.6	–
**Continuation of above table**									
**Variables**	**Characteristics**	**Point difference in diarrhea prevalence**							
		2000 N = 10,753	2005 N = 10,039	2011 N = 10,946	2016 N = 10,337	Phase1 (2005–2000)	Phase2 (2011–2005)	Phase3 2016–2011	Over all (2016–2000)
**Residence**	**Urban**	21.1	12.3	11.2	10.9	−8.8	−1.1	−0.3	− 10.2
	**Rural**	26.2	18.6	13.9	12	−7.6	−4.7	−1.9	− 14.2
**Region**	**Tigray**	17.9	12.9	13.4	13	−5	0.5	−0.4	−4.9
	**Afar**	20.6	13.5	12.7	11.5	−7.1	−0.8	−1.2	−9.1
	**Amhara**	20.3	14.7	13.7	13.8	−5.6	−1	0.1	−6.5
	**Oromia**	27.2	17.8	11.5	10.7	−9.4	−6.3	−0.8	−16.5
	**Somali**	22.8	12.4	20	6.1	−10.4	7.6	−13.9	− 16.7
	**B/Gumuz**	26.9	21.3	23.2	8.8	−5.6	1.9	−14.4	− 18.1
	**SNNP**	32.5	25.2	16.5	14	−7.3	−8.7	−2.5	−18.5
	**Gambella**	32	14.3	22.9	16	−17.7	8.6	−6.9	−16
	**Harari**	26.1	19	11.5	12.5	−7.1	− 7.5	1	−13.6
	**Addis Ababa**	16.8	13.6	9.6	7.7	−3.2	−4	−1.6	−9.1
	**Dire-Dawa**	25.7	11.8	8.3	11.6	−13.9	−3.5	3.3	−14.1
**Mother’s educational level**	**No formal education**	26.2	18.3	14	11.3	−7.9	−4.3	−2.7	−14.9
	**Primary**	24.1	19.5	12.8	13.3	−4.6	−6.7	0.5	−10.8
	**Secondary**	19.4	10.7	10.6	14.7	−8.7	−0.1	4.1	−4.7
	**Higher**	41.7	2.4	11.3	7.3	−39.3	8.9	−4	−34.4
**Father’s educational level**	**No formal education**	25	18.2	13.7	10.5	−6.8	−4.5	−3.2	−14.5
	**Primary**	29.1	19.2	14	13.4	−9.9	−5.2	−0.6	−15.7
	**Secondary**	20.8	14.7	11.6	12.8	−6.1	−3.1	1.2	−8
	**Higher**	22.1	6.3	10.6	10	−15.8	4.3	−0.6	−12.1
**Sex of household head**	**Male**	25.7	18.2	13.4	12.2	−7.5	−4.8	−1.2	−13.5
	**Female**	24.9	17.3	14.2	9.9	−7.6	−3.1	−4.3	−15
**Media exposure**	**Yes**	24.3	16.1	13.4	14	−8.2	−2.7	0.6	10.3
	**No**	25.7	18.4	13.6	11.4	−7.3	−4.8	−2.2	−14.3
**family members**	**= < 5**	27.9	18.1	13.7	10.5	−9.8	−4.4	−3.2	−17.4
	**= > 6**	23.9	18	13.4	13.6	−5.9	−4.6	0.2	−10.3
**Number of U-5 children**	**= < 2**	25.8	18.9	13.6	12.6	−6.9	−5.3	−1	−13.2
	**= > 3**	24.4	18	13.5	8.4	−6.4	−10.9	−5.1	− 16
**Drinking water**	**Improved**	25.1	16.6	12.6	12	−8.5	−4	−0.6	− 13.1
	**Not improved**	25.7	19.9	14.6	11.4	−5.8	−5.3	−3.2	−14.3
**Child waste disposal**	**Safe**	24.3	19.4	14.2	14.6	−4.9	−5.2	0.4	−9.3
	**Not safe**	25.8	17.9	13.7	10.8	−7.9	−4.2	−2.9	−15
**Type of toilet**	**Improved**	23.6	20.7	12	9.8	− 2.9	−8.7	− 2.2	−13.8
	**Not improved**	25.9	17.9	13.8	11.6	−8	−4.1	−2.2	−14.3

Regarding households’ wealth status 35% of the households in 2005, 44.7% in 2011 and 46.9% in 2016 were poor. Though Community Based Health Insurance (CBHI) was not practiced in 2000 and 2005, exactly 99.4% of the households in 2011 and 96.3% in 2016 didn’t use it. More than two third; 74.6% in 2005, 75.2% in 2011 and 60.8% of the households in 2016 reported that distance to health facility was their big problem (Table 1).

### Overall trends of diarrhea among under five-children in Ethiopia

**Fig. 1:**
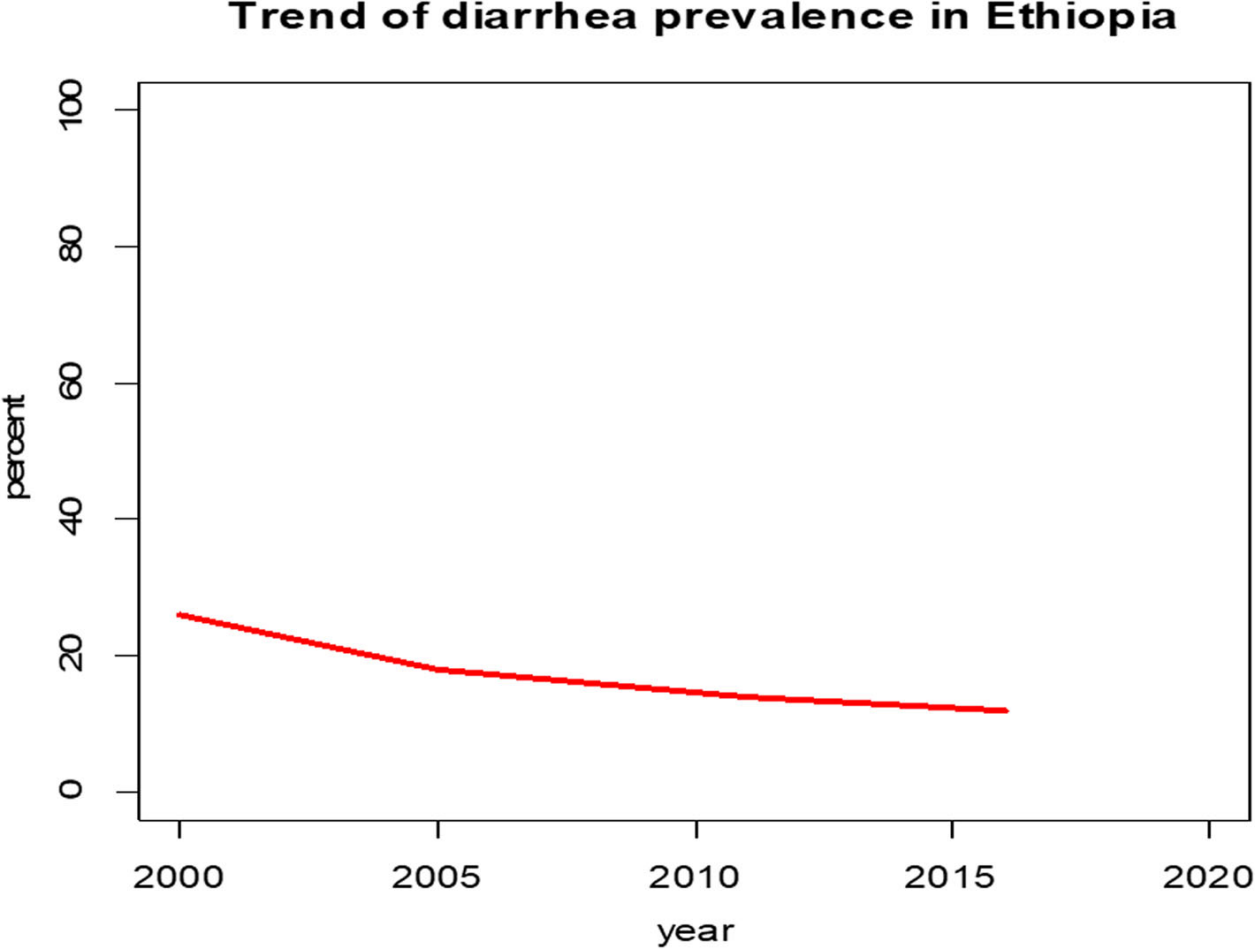
Trend of diarrhea prevalence in Ethiopia from 2000 to 2016

By looking the trend, Ethiopia has been shown a decrement in diarrhea prevalence among under-five children over the study period, from 26% in 2000 to 18% in 2005, to 14% in 2011 and to 12% in 2016. The highest decrement was noticed in the first phase (2000–2005 with a 10.4% point change compared with 8, 4 and 2% point change in second phase (2005–2011) and in the third phase (2011–2016) respectively. The overall change (2000–2016) in diarrhea prevalence was 14% (Fig. 1).

### Trends of diarrhea prevalence in Ethiopia by selected characteristics

**Fig. 2:**
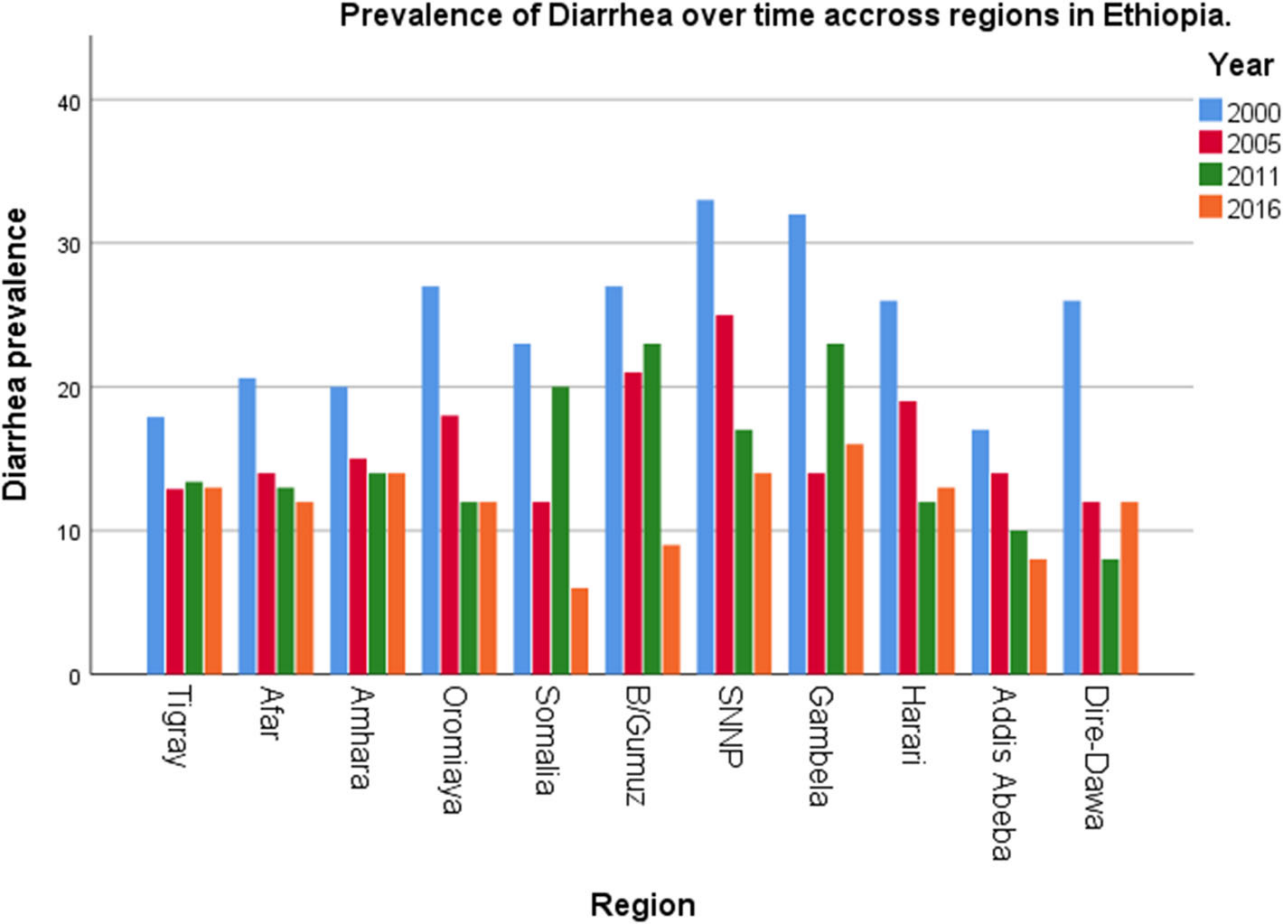
The trends of diarrhea prevalence over time across regions in Ethiopia 2000, 2005, 2011 and 2016

The trends of diarrhea prevalence among under-five children showed variation based on different characteristics. Diarrhea prevalence decrement was observed in most of the characteristics and increment in some of the characteristics in each phases. Among rural residents, the largest decrement was observed during the first phase of the study period (2000–2005) with 7.6% point change followed by second (2005–2011) and third (2011–2016) phases with 4.7 and 1.9% point changes respectively and the overall change (2000–2016) was 14.2% point change. Based on region, the largest point change in first phase was observed in Gambella regional state with 17.7% point change followed by Dire-Dawa with 13.9% point change. But in the second phase, diarrhea prevalence was increased in Gambella by 8.6% point change, in Benishangul-Gumuz by 1.9% point change and in Tigray by 0.5% point change (Fig. 2). Similarly in third phase it was increased by 3.3% point change in Dire-Dawa and by 1% point change in Harari (Fig. 2). The overall change of decrement of diarrhea prevalence based on region was higher in southern nation nationalities and people of Ethiopia (SNNP) regional state with 18.5% point change (Fig. 2). Households that have more than three under-five age children showed highest point of change (10.9%) in the second phase and the overall point change of diarrhea prevalence was 16%. Respondents who had have improved drinking water source showed decrement of diarrhea prevalence among under-five children with 8.5, 4, 0.6 and 13.1% point change in first, second, third and fourth phases respectively (Table 2).

### Decomposition analysis

Overall from 2000 to 2016, there has been a significant decline in prevalence of diarrhea in Ethiopia. The overall decomposition result showed that 97.1% of decline in prevalence of diarrhea over time was due to behavioral changes between the surveys. About 2.9% of decline was due to difference in characteristics (population proportion) but the change due to difference in characteristics (population proportion) was not significant (Table 3).

**Table 3:** Decomposition of change in diarrhea prevalence among under-five children in Ethiopia, from 2000 to 2016

Diarrhea	Coef	95% CI		Pct.	
		L-CI	U-CI		
**E**	−.004	−0.016	0.008	2.9	
**C**	−0.13	−0.15	− 0.11	97.1*	
**R**	−0.14	−0.2	− 0.11*		

**Variables**	**Characteristics**	Difference due to characteristics		Difference due to coefficient	
		Coef	Pct.	Coef	Pct.
**Residence**	**Urban (ref)**				
	**Rural**	0.000 (−0.00.., 0.00)	−0.05	− 0.000(− 0.06, 0.6)	0.62
**Mothers work status**	**Had work (ref)**				
	**Had no work**	−0.003 (− 0.01, 0.005)	2.47	− 0.02*(− 0.05,-0.003)	14
**Mother’s educational level `**	**No formal (ref) education**				–
	**Primary**	−0.0001 (− 0.002, 0.002)	0.11	0.002(− 0.004, 0.008)	−1.6
	**Secondary**	0.000(0.000, 0.0000)	−0.000	0.001(−0.003, 0.006)	−3.1
	**Higher**	−0.001(− 0.002,0.001)	0.5	− 0.001* (− 0.001, − 0.000)	0.4
**Father’s educational level**	**No formal (ref)** **education**				
	**Primary**	0.002 (− 0.001, 0.005)	−1.3	0.001(− 0.009, 0.01)	− 0.7
	**Secondary**	−0.0001 (− 0.0006, 0.000)	0.1	0.004(− 0.02, 0.01)	− 3.1
	**Higher**	0.000 (− 0.001, 0.0010	− 0.13	0.001(− 0.001, 0.003)	−0.63
**Family members**	**<=5 (ref)**				
	**> = 6**	−0.002 (− 0.005, 0.001)	1.2	0.41*(0.02, 0.06)	−30
**Number of under-five children**	**<=2 (ref)**				
	**> = 3**	− 0.001 (− 0.002, 0.000)	0.47	− 0.005(− 0.011, 0.002)	3.7
**Sex of household head**	**Male (ref)**				
	**Female**	0.0003 (−0.000, 0.001)	−0.22	− 0.004(− 0.1, 0.003)	2.6
**Media exposure**	**Yes (ref)**				
	**No**	0.001(−0.008, 0.003)	− 0.87	− 0.02(− 0.07, 0.03)	16.7
**Water source**	**Improved (ref)**				
	**Not improved**	0.000 (− 0.004, 0.004)	− 0.005	0.04(− 0.017, 0.046)	−10.4
**Constant**				−0.15* (− 0.23, − 0.06)	106.1

(* = significant at 5% level of significance), (Coef = coefficient),(CI = confidence Interval),(L-CI = lower confidence interval),(U-CI = upper confidence interval), (Pct = percent), (**E =** Difference due to characteristics), (**C** = Difference due to coefficient), (**R** = over all difference)

Factors including mother’s education level, number of family members and mothers working status showed a significant effect for the decline of diarrhea prevalence. Keeping compositional changes constant, change in behavior of mothers who have higher education level contributed 0.4% for the decline of diarrhea prevalence for the last sixteen years as compared to mothers who had no formal education. Compared with mothers who had work, behavioral change of mothers who had no work contributes 14% for decrement of diarrhea prevalence over time. Similarly, behavioral change of respondents who have more than six family members contributed 30% for the decline of diarrhea prevalence for the last sixteen years as compared to respondents who have less than five family members (Table 3).

### Multilevel analysis based on Bayesian approach

#### Model with both individual and community level factors

As shown in Table 4, this model Rhat value is one and all effective sample sizes (both Bulk_ESS and Tail_ESS) are greater than 1000. Therefore this model was converged. This model has smallest Widely Applicable Information Criteria (WAIC =7904) as compared to random intercept only model (WAIC = 74,976), model with only individual level factors (WAIC = 7981) and model with only community level factors (WAIC = 7958). Therefore this model is the best fitted model for the data because it has smallest WAIC as compared to the rest models. So interpretation and reports were made based on this model. Of all the factors included in the full model (model with both individual and community level factors) for multilevel analysis, being twin, child’s age, weight of child at birth, vaccinated for measles and rotavirus, number of under-five children, number of family members, wealth index, distance to health facility, member of health insurance and child waste disposal method were significantly associated with under-five children diarrhea in Ethiopia.

**Table 4:** Relationship analysis for diarrhea and individual and community level factors based on Bayesian approach in Ethiopian under-five children in 2016

Fixed effect	Category	Estimates	SE	AOR	95%CrI of AOR		Rhat	Bulk_ ESS	Tail_ ESS
					L-CrI	U-CrI			
훽0 intercept*		−5.8	0.7	0.00	0.00	0.01	1	5814	9217
**Sex of child**	**Male (ref)**								
	**Female**	−0.11	0.07	0.89	0.78	1.02	1	10,361	5807
**Twin**	Yes*	0.26	0.07	1.3	1.1	1.5	1	19,613	8366
	**No (ref)**								
**Weight of child at birth**	**Small**	−0.19	0.11	0.83	0.67	1.02	1	19,808	14,137
	**Average (ref)**								
	Big*	0.48	0.11	1.63	1.62	2.02	1	20,612	13,727
**Birth order**	**First (ref)**								
	**2–3**	−0.36	0.26	0.7	0.43	1.2	1	10,903	11,827
	**4–5**	− 0.25	0.29	0.78	0.44	1.38	1	10,235	10,907
	**= > 6**	−0.29	0.3	0.76	0.42	1.35	1	10,209	10,942
**Birth interval**	**= < 23 month**	−0.03	0.08	0.97	0.83	1.13	1	20,381	13,298
	**= > 24 month (ref)**								
**Age of child**	**< 1 year (ref)**								
	= > 1 < 2 year*	0.22	0.08	1.3	1.06	1.47	1	9648	5850
	**= > 2 < 3 year**	− 0.15	0.09	0.86	0.72	1.03	1	3604	4530
	= > 3 < 4 year*	−0.54	0.1	0.6	0.48	0.71	1	3251	4625
	**= > 4 < 5 year**	−1.23	0.12	0.3	0.23	0.4	1	3377	4837
**Breast feeding**	**Still breast feed (ref)**								
	**Ever breast feed but not now**	−0.09	0.8	0.91	0.78	1.07	1	24,022	13,505
	**Never breast feed**	−0.27	0.31	0.80	0.42	1.42	1	22,263	12,015
**Rota vaccine**	**vaccinated (ref)**								
	Not vaccinated*	0.32	0.13	1.44	1.12	1.9	1	9367	6075
**Measles’ vaccine**	**Yes (ref)**								
	No*	0.2	0.05	1.2	1.1	1.33	1	14,128	6067
**Vitamin supplementation**	**Yes (ref)**								
	**No**	−0.12	0.06	0.88	0.78	1.01	1	25,507	14,935
**Wealth index**	Poor*	.95	0.23	2.6	1.7	4.03	1	16,045	14,275
	**Medium**	0.09	0.24	1.1	0.68	1.74	1	14,461	14,475
	**Rich (ref)**								
**Mothers working status**	**Working (ref)**								
	**Not working**	0.24	0.2	1.3	1.0	1.6	1	8311	5878
**Health insurance**	**Yes (ref)**								
	No*	0.78	0.27	2.2	1.3	3.8	1	7888	5393
**Continuation of above table**									
**Fixed effect**	**Category**	Estimates	SE	AOR	95%CrI of AOR		Rhat	Bulk_ ESS	Tail_ ESS
					L-CrI	U-CrI			
**Distance to health facility**	**Not long (ref)**								
	Long *	1	0.12	2.7	2.2	3.5	1	6206	5558
**Mother’s educational level**	**No formal education (ref)**								
	**Primary**	0.1	00.1	1.1	0.8	1.4	1	8748	10,747
	**Secondary**	0.1	00.3	1.1	0.6	1.9	1	11,355	12,382
	**Higher**	−0.5	0.36	0.6	0.1	1.3	1	7021	1091
**Health insurance**	**Yes (ref)**								
	No*	0.78	0.27	2.2	1.3	3.8	1	7888	5393
**Distance to health facility**	**Not long (ref)**								
	Long *	1	0.12	2.7	2.2	3.5	1	6206	5558
**Father’s educational level**	**No formal education (ref)**								
	**Primary**	0.3	0.1	1.3	0.99	1.7	1	9386	12,115
	**Secondary**	0.25	0.2	1.3	0.8	1.9	1	8416	10,409
	**Higher**	0.1	0.3	1.1	0.6	2	1	9987	10,442
**Household head**	**Male (ref)**								
	Female*	−0.22	0.13	0.81	0.61	1.05	1	8669	6074
**Media exposure**	**Yes (ref)**								
	**No**	0.27	0.19	1.30	0.89	1.9	1	17,430	14,082
**family members**	**= < 5(ref)**								
	= > 6*	0.35	0.13	1.41	1.1	1.83	1	7623	5854
**No of under five children**	**= < 2 (ref)**								
	= > 3*	0.26	0.11	1.3	1.1	1.61	1	9026	5453
**Source of drinking water**	**Improved (ref)**								
	**Not improved**	−0.17	0.12	0.84	0.66	1.06	1	19,868	13,216
**Child waste disposal**	**Safe (ref)**								
	Not safe*	1.44	0.14	4.2	3.2	5.6	1	7238	5718
**Type of toilet**	**Improved (ref)**								
	**Not improved**	0.21	0.13	1.23	0.95	1.6	1	18,719	13,886
**Random effect**									
${{\boldsymbol{\sigma}}_{{\boldsymbol{\mu}}_{\mathbf{0}}}}^{\mathbf{2}}$		0.36	0.05		0.25	0.52	1	1500	2913
**ICC**		0.11			0.10	0.13			
**LOO**		7931							
**WAIC**		7904							

(Ref = reference category), (* = significant at 5% level of significance), (SE = standard error), (CrI = credible interval), (L-CrI = lower credible interval), (U-CrI = Upper credible interval)

Being twin, the odds of having diarrhea were 30% (AOR = 1.3; 95% CrI 1.1–1.5) higher than those children who were single (AOR = 1.3; 95% CrI 1.1–1.5). The odds of having diarrhea among children whose weight was big at birth were 63% (AOR = 1.63; 95% CrI 1.62–2.02) higher as compared to children whose weight was average (normal) at birth. The odds of developing diarrhea among children in the age group between 1 and 2 years were 1.3 times (AOR = 1.3; 95% CrI 1.06–1.47) higher than those children whose age was below one years. Children who were not vaccinated for rotavirus and Measles were 1.44 and 1.2 times (AOR = 1.44; 95% CrI 1.12–1.9, AOR = 1.2; 95% CrI 1.1–1.33) more likely to develop diarrhea than to those who were vaccinated for rotavirus and measles respectively. The odds of developing diarrhea in children living in households who were not a member of health insurance were 2.2 times (AOR 2.2; 95% CrI 1.3–3.8) higher than children living in households who a member of health insurance. And also children living in households who travel long distance to health facility were 2.7 times (AOR 2.7; 95% CrI 2.2–3.5) higher than children living in households who short distance to health facility. The odds of having diarrhea among children living in households with no safe child waste disposal methods were 4.2 times (AOR 4.2; 95% CrI 3.2–5.6) higher than in children living in households with safe child waste disposal methods (Table 4).

## Discussion

Diarrheal diseases are a major cause of children mortality and one of the main causes of medical consultation for children in Sub-Saharan African countries [12].

In this study, the trend of diarrhea prevalence has been significantly declined from 26% in 2000 to 12% in 2016 (overall phase). This finding is compatible with the study done in democratic republic of Congo [8, 25]. This figure might be due to the launching of the Health Extension Program (HEP), improving access to health care to meet the primary attention of the MDG agenda and the introduction of integrated community cause management program [26, 27]. When we decompose this change, behavioral change of the respondents between the surveys contributed 97.1% for the decline of diarrhea prevalence over the last sixteen years. From decomposition analysis, behavioral change of women who had higher education level contributed 0.4% for the change of diarrhea prevalence among under-five children in Ethiopia. Similarly, behavioral change of women who were not working and households who had more than six family members contributed 14 and 30% respectively for the change. Comparable finding was also reported from the study conducted in Democratic Republic of Congo [25]. This finding could be due to Governments commitment to improve awareness of the community through health education and enabling them to use health services.

The multilevel binary logistic regression analysis based on Bayesian approach reveled that from child socio-demographic characteristics; being twin, weight of the child at birth and age of the child were significantly associated with diarrhea among under-five children. As indicated by related literatures; similarly, this finding showed that being twin were more risk to have diarrhea as compared to children who were single. This finding is consistent with the study conducted in Bangladesh, Cameroon, Nigeria and Niger [28–30]. This might be due to children who are twin might not get exclusive breast milk at early ages and this reduce their immunity and prone to diarrhea. Similarly, the quality of care and attention from parents decreased. So they are easily susceptible for different diseases. Children who were obese at birth were more likely to develop diarrhea as compared to children who were normal at birth. This might be due to microbial metabolites, particularly short chain fatty acids, can lead to signaling changes in the host enterocytes and motility disorders and finally causes diarrhea [11]. The odds of developing diarrhea among children in the age group between 1 and 2 years were higher than those children whose age was below one year. On the contrary, the odds of developing diarrhea among children in the age group between 3 and 4 years were less likely to be occur than those children whose age was below one year. This finding was supported by previous studies conducted from Ethiopia, Ghana, Cameroon, Bangladeshi, Niger and Nigeria [31–35]. This could be due to, the age six month to two years are the time of crawling and at this time children eat whatever they get even their fecal matter if their care givers are irresponsive for their child care; but children whose age is greater than two years can differentiated dirty things and don’t eat whatever they get. In addition to this, even though children often are breastfed until 1 year old and lower chance of drinking contaminated water and developing diarrhea, an immune system of a 3–4 year old is already more developed and thus acquired better immunity compared to a 1 year old children.

From Socio-economic and demographic characteristics of household; number of under-five children, family size, wealth status of the household, member of health insurance and distance to health facility were significantly associated with diarrhea among under-five children. Children from households who had greater than two under-five children were more risk to experience diarrhea as compared to children from households who had equal to or less than two under-five children. Similarly, children who were from households who had greater than six family members were more risk to develop diarrhea as compared to children who were from households who had less than five family members. This finding is concurrent with previous studies conducted in Ethiopia [34, 36, 37]. If the number of under-five children and family members increased in the household, it is expected that children will be more vulnerable to diarrhea mainly because of the decreased quality of care and attention from parents. The odds of having diarrhea in children who were from poor household wealth status were higher than those who were from the rich households. This finding is supported by the study conducted in India [29, 30]. This is because in resource-limited settings, like Ethiopia; children can’t get balanced diets, improved type of drinking water and health care [38]. The odds of developing diarrhea among children living in households who were not a member of health insurance and who travel long distance to health facility was higher as compared to children living in households who were a member of health insurance and who travel short distance to health facility. This study was supported by a study conducted in Egypt, Nigeria and Tanzania [28, 30, 39]. This might be due to the fact that households who hadn’t health facilities close to their area of residence and who have not community based health insurance may not access health care services easily.

Moreover, from the child care related factors, vaccinated for rotavirus and measles were associated with diarrhea among under five children. The study revealed that children who were not vaccinated for rotavirus and measles were more risk to experience diarrhea as compared to those children who were vaccinated. This finding was in agreement with the previous study conducted in Ethiopia, [40]. Measles is a highly contagious disease which disrupts the epithelial cells and suppresses the immune system leading to infection in various organ systems and protein losing enteropathy [41]. Similarly, Rotavirus is the most common cause of severe gastroenteritis and diarrhea among young children worldwide [42]. Due to this reason, rotavirus vaccine was introduced by WHO in 2006 [43] and after 7 years, Ethiopia has begun to give rotavirus vaccine in 2013 [38]. Once more, from the hygiene and sanitation related factors, unsafe disposal of child waste was significantly associated with diarrhea among under five children. Children who were from households who dispose waste unsafely were more likely to develop diarrhea as compared to children who were from households who dispose waste safely. This finding is consistent with the findings in Bangladeshi and sub-Saharan countries (Nigeria, Niger, and Burkina Faso) [31–35]. This is because if they don’t disposed any waste materials properly including child’s wastes, children as well as adults are risk for feco-oral diseases through flies [44].

### Strengths and limitations of the study

Fitting multilevel model using Bayesian approach to get fine estimates of the parameters and considering all the national regional states of Ethiopia by taking large sample size at different time points was the strength of this study. As a limitation we can’t get some variables for each survey (for example health insurance, distance to health facility and wealth index) to show trends and to perform decomposition analysis.

## Conclusion and recommendations

The prevalence of diarrhea was significantly declined over the last sixteen years and the decline was due to behavioral changes between the surveys. A major driver for decline in diarrhea prevalence over time was behavioral change of respondents who have more than six family members and contributed 30% for the decline. Based on multilevel analysis being twin, age of the child, weight of child at birth, vaccinated for measles and rotavirus, number of under-five children, number of family members, wealth status, distance to health facility, health insurance and child waste disposal method were significantly associated with diarrhea among under five-children in Ethiopia. Therefore Ethiopian government and Ministry of Health should focus on the strengthening and scaling up of behavioral change package strategies of the community regarding to keeping hygiene and sanitation of the community and their environment, vaccinating their children, accessing health care services to prevent diarrheal disease. Similarly the government should resolve structural related problems that precipitate diarrheal disease of under-five children. The Health Institutions should enforce the communities to implement diarrhea management strategies via the existing health extension packages. And also family members should be a member of health insurance, should vaccinate their children based on the national guideline, should practice safe waste disposal methods and implement all the components of health extension packages based on health professional’s order.

## Data Availability

The EDHS data sets are open and can be accessed from the Measure DHS website (http://www.dhsprogram.com) through an online request by explaining the objective of the study. The datasets analyzed during the current study are available from the corresponding author upon reasonable request.

## Abbreviations

AOR: Adjusted Odds Ratio
C: Difference due to coefficient
CrI: Credible interval
DHS: Demographic Health Survey
E: Difference due to characteristics
EA: Enumeration Area
EDHS: Ethiopian Demographic and Health Survey
HMC: Hamiltonians Monte Carlo
SE: Standard Error
L-CrI: Lower Credible Interval
LOO: Leave-One-Out Cross-Validation
Pct: Percent
R: over all difference
U-CrI: Upper Credible Interval
WAIC: Widely Applicable Information Criteria
WHO: World Health Organization
